# Clinicopathological features of 70 desmoid-type fibromatoses confirmed by β-catenin immunohistochemical staining and *CTNNB1* mutation analysis

**DOI:** 10.1371/journal.pone.0250619

**Published:** 2021-04-29

**Authors:** Jiyeon An, Ha Young Woo, Younghan Lee, Hyo Song Kim, Juhyeon Jeong, Sang Kyum Kim

**Affiliations:** 1 Department of Pathology, Severance Hospital, Yonsei University College of Medicine, Seoul, Republic of Korea; 2 Department of Radiology, Severance Hospital, Yonsei University College of Medicine, Seoul, Republic of Korea; 3 Division of Medical Oncology, Department of Internal Medicine Severance Hospital, Yonsei University College of Medicine, Seoul, Republic of Korea; 4 Department of Pathology, Gachon University Gil Medical Center, Incheon, Republic of Korea; University of Milan Bicocca, ITALY

## Abstract

Desmoid-type fibromatosis (DF) is a locally aggressive neoplasm characterized by mutations in the *CTNNB1* gene, which encodes the β-catenin protein. We reviewed 85 cases of DF and performed Sanger sequencing for detecting mutations in *CTNNB1* and immunostaining for detecting β-catenin localization. We included 70 DF samples, of which 56 cases demonstrated nuclear β-catenin localization and 43 cases harboured *CTNNB1* mutations. *CTNNB1-*mutant DF samples consistently displayed nuclear β-catenin expression and were derived from larger-sized tumours compared to samples with wild-type *CTNNB1*. When we further classified DF cases into 2 subgroups based on the type of specimen, excised specimens with nuclear β-catenin expression frequently displayed *CTNNB1* mutation and no statistical correlation between nuclear β-catenin expression and *CTNNB1* mutation was observed in biopsies. When we classified *CTNNB1* mutation cases into 2 subgroups (DF with T41A or T41I, and DF with S45F or S45P), T41A or T41I mutations were observed more frequently in males than in females. Additionally, DF tumours harbouring S45F or S45P mutations were located more frequently in the abdominal wall than tumours with T41A or T41I mutations. In conclusion, *CTNNB1* mutation correlates with nuclear β-catenin expression in larger or excised DF tumours, and DF harbouring *CTNNB1* mutations manifest variable clinical presentations.

## Introduction

Desmoid-type fibromatosis (DF; also called desmoid tumour, deep fibromatosis, or aggressive fibromatosis) is a borderline soft tissue tumour that displays variable aggressiveness and a high proclivity for recurrence [1,2]. Because of the high recurrence rate and preventable morbidity associated with repeated surgeries, the treatment options for DF vary from surgery to watchful waiting with careful follow-up [2–4]. DF tumours are classified based on their location as tumours in the abdominal wall, intra-abdominal cavity, or extra-abdominal sites. It is well known that up to 80% of sporadic lesions, regardless of tumour location, harbour mutations in the *CTNNB1* gene, which encodes the β-catenin protein [5]. The most common *CTNNB1* mutations in DF are observed in exon 3 and include T41A, S45F, and S45P [1,6]. A novel pathogenic alteration in DF, the T41I mutation, was recently reported [7].

When mutations occur, β-catenin degradation is impaired in the cytoplasm, leading to its accumulation in the nucleus [6,8–10]. Nuclear beta-catenin staining was seen in a very limited subset of sof tissue tumor types, including desmoid-type fibromatosis, solitary fibrous tumor, endometrial stromal sarcoma and synovial sarcoma [11]. β-catenin is expressed in approximately 70−75% of DF and is used to differentiate DF from postoperative scar formation caused by other soft tissue tumours such as fibroblastoma [12]. Therefore, nuclear staining using an antibody against β-catenin is used as a marker for diagnosing DF [6,13].

Smooth-muscle actin (SMA) is usually expressed in DF, but is an unspecific marker and is not expressed in all cases of DF [12,14,15]. In diagnostic practice, it is hard to interpret subcellular β-catenin expression in the biopsied specimens or in small-sized tissues because tumour cells stain variably for β-catenin. Furthermore, genetic studies for identifying *CTNNB1* mutations cannot be performed for all specimens used for the diagnosis of DF in pathology laboratories. Further investigation is required to determine whether immunohistochemical staining for β-catenin in different specimen types and in varying tumour sizes is indicative of mutations in the *CTNNB1* gene.

In this study, we reviewed 85 cases of pathologically diagnosed DF and performed β-catenin immunohistochemical staining and *CTNNB1* gene sequencing on tumour samples. Furthermore, we compared the β-catenin expression with *CTNNB1* mutation status and analysed the clinicopathological features according to the molecular subtypes of DF and the types of specimens.

## Materials and methods

### Patients and samples

**Fig 1:**
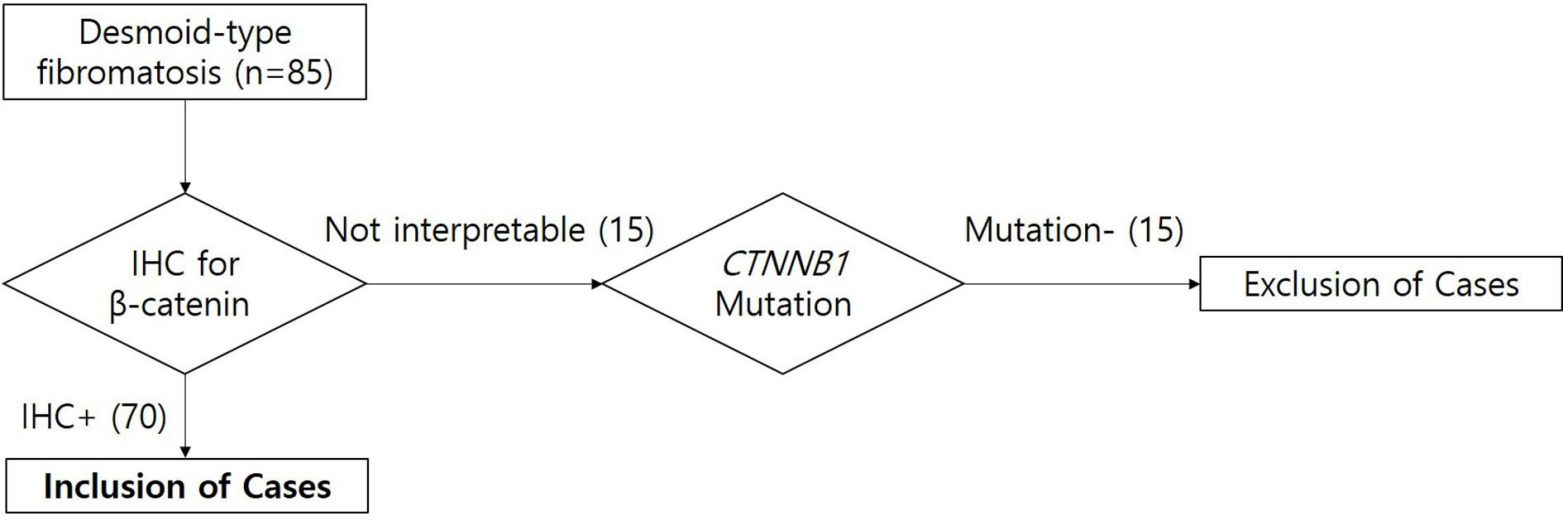
The algorithm for the selection of eligible cases of DF in this study. IHC, immunohistochemistry.

We retrospectively recruited 85 patients who were diagnosed with DF from 2005 to 2013 at Severance Hospital, Yonsei University College of Medicine. Medical records were reviewed for clinical features and radiological and pathological findings. To select the most representative formalin-fixed paraffin-embedded (FFPE) tissues for immunohistochemical staining and molecular studies, samples were mounted on slides, stained with haematoxylin and eosin, and reviewed by two pathologists (JH Jung & SK Kim). The algorithm for the selection of eligible cases of DF in this study is shown in Fig 1. We excluded the uninterpretable cases which did not show internal positive control staining of β-catenin by immunohistochemistry. All the uninterpretable cases had wild type *CTNNB1* genes in Sanger sequencing. Finally, 70 specimens of DF were included. Preoperative radiological imaging was available for 64 patients.

All methods and experimental protocols using human tissues were carried out in accordance with relevant guidelines and regulations approved by the Institutional Review Board of Severance Hospital, Yonsei University Health System, and the patients’ consent form were waived (IRB no. 4-2016-0687).

### Immunohistochemistry

FFPE blocks were cut into 4-μm-thick sections and processed using heat-induced epitope retrieval. Immunohistochemical staining was performed in an automated immunostainer (Ventana Discovery® XT, Ventana Medical System, Inc., Oro Valley, AZ, USA). Antibodies against β-catenin (1:400, Cell Marque, #224ㅡ15) and SMA (1:1000, DAKO, #M0851) were utilized. Two pathologists (JY Ahn & JH Jung) blinded to the pathologic information independently evaluated the immunohistochemical staining. β-catenin staining was classified as negative or positive according to the subcellular staining pattern. Cytoplasmic, membranous, or Golgi staining pattern was regarded as negative expression and nuclear staining as positive expression when observed in over 1% of tumour cells. We regarded positive expression for SMA when it displayed cytoplasmic expression in more than 1% of the tumour area. We defined negative or positive staining regardless of staining intensity for both beta-catenin and SMA.

### DNA extraction and sequencing

Polymerase chain reaction was conducted to evaluate *CTNNB1* mutation status. Genomic DNA was extracted from 10-μm sections cut from FFPE tissue blocks using the Maxwell^®^ CSC DNA FFPE extraction kit (Promega, Madison, WI, USA) and the Maxwell^®^ CSC instrument. Polymerization was performed with the primers *CTNNB1* ex3F (forward, 5’-TTTGATGGAGTTGGACATGG-3’) and ex3R (reverse, 5’-GAAGGACTGAGAAAATCCCTGTT-3’), to amplify exon 3 of *CTNNB1*. The polymerization reaction was performed using Taq polymerase (BIOLINE, London, UK), with 45 cycles of denaturation (94°C, 30 sec), annealing (65°C, 30 sec), and polymerization (72°C, 30 sec). After purification by electrophoresis using a 2% agarose gel containing ethidium bromide, analysis was conducted using the Pyromark Q24 2.0.7 Software.

### Statistical analysis

All statistical analyses were performed using SPSS version 18.0 (IBM, Chicago, IL, USA) and GraphPad Prism version 7.04 (GraphPad software, San Diego, CA, USA). The relationships between groups were compared using the χ2 test, Fisher’s exact test, or unpaired t-test. Two-sided *P* values < 0.05 were considered statistically significant.

## Results

### Clinical features of DFs

We reviewed 85 cases of pathologically diagnosed DF and performed immunohistochemical staining for ß-catenin and *CTNNB1* gene mutation tests on all the samples (Fig 1). Nuclear ß-catenin expression was identified in 56 out of 85 DF samples, whereas *CTNNB1* gene mutation was observed in 43 out of 85 DF samples. Among the 85 cases of DF, we excluded 15 cases which did not show internal positive control staining of β-catenin and these concurrently displayed negative ß-catenin staining and a wild-type *CTNNB1* gene. Thus, we excluded these ß-catenin-negative and *CTNNB1*-wild type cases for further analyses.

**Fig 2:**
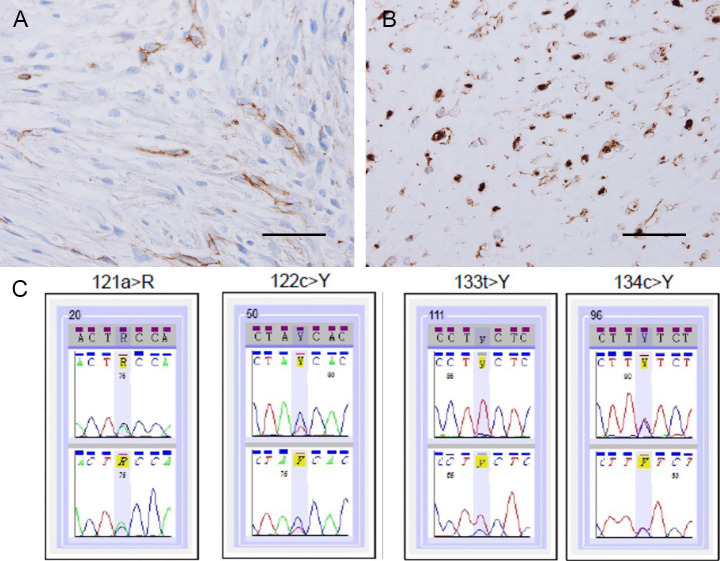
Immunohistochemical staining for β-catenin and identification of *CTNNB1* gene mutation. A, Cytoplasmic expression of β-catenin (negative). B, Nuclear expression of β-catenin (positive). C, *CTNNB1* gene mutation in exon3 codon 41 (121A>G, T41A; 122C>T, T41I) and codon 45 (133T>C, S45P; 134C>T, S45F). Scale bar, 50μm.

Next, we analysed clinicopathological features of 70 patients with DF (Table 1). All patients were between 2 months and 84 years old (mean age: 36.01 ± 20.89 years) with 29 males and 41 females. Nine cases manifested in the abdominal wall (12.86%), 9 in the abdominal cavity (12.86%), and 52 in extra-abdominal sites (74.29%). Tumour sizes were measured during preoperative imaging and ranged from 0.9 cm to 17.0 cm in size (mean size: 6.733 ± 4.424 cm). Sixty tumours were excised and 10 cases were biopsied. Forty-three cases out of 60 patients from whom tumours were excised underwent follow-up and 19 cases of recurrence were observed (44.19%). We performed immunohistochemical staining for ß-catenin and SMA and observed nuclear ß-catenin expression in 56 cases (80.0%; Fig 2) and SMA positivity in 61 cases (87.14%), which were found to be consistent with the literature [5]. There was no statistical difference between ß-catenin and SMA expressions in DFs (S1 Table).

**Table 1:** Clinicopathological parameters in desmoid-type fibromatosis patients.

Clinicopathological Parameters (*n* = 70)	*n* (%)
**Sex**	
** Male**	29 (41.4)
** Female**	41 (58.6)
**Age (years; mean ± SD)**	36.01 ± 20.89
**Site**	
** Abdominal wall**	9 (12.9)
** Abdominal cavity**	9 (12.9)
** Extra-abdominal sites**	52 (74.3)
**Size (cm, mean ± SD)**	6.733 ± 4.424
**Specimen types**	
** Biopsy**	10 (14.3)
** Excision**	60 (85.7)
**Recurrence (N = 43)**	19 (44.2)
**β-catenin expression**	
** Positive**	56 (80.0)
** Negative**	14 (20.0)
**SMA expression**	
** Positive**	61 (87.1)
** Negative**	9 (12.9)

SD, standard deviation; SMA, smooth muscle actin.

### *CTNNB1* mutations in desmoid-type fibromatosis

Using Sanger sequencing, we detected *CTNNB1* mutations in 43 out of 70 patients (61.43%; Fig 2C). We divided DF patients into 2 groups according to ß-catenin expression and CTNNB1 mutation status — those with ß-catenin expression or *CTNNB1* mutations and those without ß-catenin expression and *CTNNB1* mutations — and compared clinical features between the groups (S2 Table). There was no significant statistical difference in sex, age at diagnosis, tumor site, tumor size, procedures, recurrence and SMA expression between the groups.

Next, we divided DF patients into 2 groups — those with *CTNNB1* mutations and those without *CTNNB1* mutations — and compared clinical features between the groups (Table 2).

**Table 2:** Clinicopathological findings in desmoid-type fibromatosis patients according to *CTNNB1* mutation status.

	*CTNNB1* Wild-type (*n* = 27)	*CTNNB1* Mutation (*n* = 43)	*p-*value
**Sex**			0.804
**Male**	12 (44.4)	17 (37.0)	
**Female**	15 (55.6)	26 (63.0)	
**Age at diagnosis (years; mean ± SD)**	31.93 ± 3.653	33.29 ± 5.642	0.099
**Site**			0.869
**Abdominal wall**	3 (11.1)	6 (14.0)	
**Abdominal cavity**	3 (11.1)	6 (14.0)	
**Extra-abdominal**	21 (77.8)	31 (72.1)	
**Size (cm; mean ± SD)**	4.973 ± 0.952, n = 22	7.655 ± 0.642, n = 42	0.020
**Specimen**			0.493
**Biopsy**	5 (18.5)	5 (11.6)	
**Excision**	22 (11.5)	38 (88.4)	
**Recurrence (n = 43)**			0.349
**Absent**	10 (66.7)	14 (50.0)	
**Present**	5 (33.3)	14 (50.0)	
**β-catenin expression**			0.012
**Negative**	10 (37.0)	4 (9.3)	
**Positive**	17 (63.0)	39 (90.7)	
**SMA expression**			0.079
**Negative**	6 (22.2)	3 (7.0)	
**Positive**	21 (77.8)	40 (93.0)	

Values are expressed as *n* (%) unless otherwise specified. SD, standard deviation; SMA, smooth muscle actin.

The results demonstrated that DF cases harbouring *CTNNB1* mutations were derived from larger-sized tumours (7.655 ± 0.642 cm) compared with DF cases without *CTNNB1* mutation (4.973 ± 0.952 cm; *p* = 0.020). Immunostaining analysis found that ß-catenin nuclear expression was more frequent in DF cases harbouring *CTNNB1* mutation (39/43, 90.70%) than in DF cases without *CTNNB1* mutation (17/27, 62.96%; *p* = 0.012). There were no significant differences between the two groups with respect to gender, age at diagnosis, tumour site, specimen type or recurrence.

No statistical differences in ß-catenin expression (p = 0.674) and CTNNB1 mutation (p = 0.493) were observed between biopsied tissues (n = 10, 14.29%) and excised tissues (n = 60, 172 85.71%) (S3 Table). We further analysed the correlation between ß-catenin expression and CTNNB1 mutation according to the specimen types (Table 3). Excised specimens demonstrated nuclear ß-catenin expression more frequently in DF cases CTNNB1 mutated (34/38, 89.47%) than in CTNNB1 wild type tumours (13/22, 178 59.09%; p = 0.009). On the contrary, there was no correlation between ß-catenin expression and CTNNB1 mutation in the biopsy specimens (p > 0.999).

**Table 3:** β-catenin expression and *CTNNB1* mutation status in desmoid-type fibromatosis patients according to specimen types.

Specimen type	β-catenin expression	*CTNNB1* status, *n* (%)		*p*-value
		Wild-type	Mutation	
**Total (*n* = 70)**	**Negative**	10 (37.0)	4 (9.0)	0.012
	**Positive**	17 (63.0)	39 (91.0)	
**Excision (*n* = 60)**	**Negative**	9 (40.9)	4 (10.5)	0.009
	**Positive**	13 (59.1)	34 (89.5)	
**Biopsy (*n* = 10)**	**Negative**	1 (20.0)	0 (0.0)	>0.999
	**Positive**	4 (80.0)	5 (100)	

### Clinicopathological features of desmoid-type fibromatosis according to *CTNNB1* mutation type

We detected *CTNNB1* mutations in 43 out of 70 DF tumours (Table 4). Twenty-seven cases displayed the T41A mutation (62.79%), 1 harboured the T41I mutation (2.33%), 12 cases harboured the S45F mutation (27.91%), and 2 cases harboured the S45P mutation (4.64%). One patient harboured both T41A and S45F mutations in the tumour.

**Table 4:** Mutation types of the *CTNNB1* gene.

*CTNNB1* gene status	*n* (%)
**Wild-type**	27 (38.56)
**Mutation**	43 (61.43)
**T41A**	27 (62.79)
**T41I**	1 (2.33)
**S45F**	12 (27.91)
**S45P**	2 (4.64)
**T41A & S45F**	1 (2.33)

After excluding the case of DF harbouring both T41A and S45F mutations, we divided the remaining 42 DF patients with *CTNNB1* mutation into two groups — those with the T41 mutation and those with the S45 mutation (Table 5). The T41 mutation occurred more frequently in males (14/16, 87.50%) than in females (14/26, 83.85%; *p* = 0.042). The S45 mutation occurred more frequently in the abdominal wall (5/14, 35.71%) than the T41 mutation (1/28, 3.57%; *p* = 0.008). However, there were no significant differences between the two groups with respect to gender, age at diagnosis, tumour size, recurrence, and β-catenin or SMA expression.

**Table 5:** Clinicopathological findings in desmoid-type fibromatosis patients according to *CTNNB1* mutation types.

*CTNNB1* Mutation Type	T41A or T41I (*n* = 28)	S45F or S45P (*n* = 14)	*p*-value
**Sex**			0.042
**Male**	14 (50.0)	2 (14.3)	
**Female**	14 (50.0)	12 (85.7)	
**Age (years; mean ± SD)**	31.93 ± 3.653	33.29 ± 5.642	0.836
**Site**			0.008
**Abdominal wall**	1 (3.6)	5 (35.7)	
**Abdominal cavity**	6 (21.4)	0 (0.0)	
**Extra-abdominal sites**	21 (75.0)	9 (64.3)	
**Size (cm; mean ± SD)**	7.675 ± 0.8431	7.854 ± 1.021	0.901
**Recurrence**			0.237
**Absent**	11 (61.1)	3 (30.0)	
**Present**	7 (38.9)	7 (70.0)	
**β-catenin expression**			0.100
**Negative**	1 (3.6)	3 (21.4)	
**Positive**	27 (96.4)	11 (78.6)	
**SMA expression**			0.545
**Negative**	2 (7.1)	0 (0.0)	
**Positive**	26 (92.9)	14 (100.0)	

Values are expressed as *n* (%) unless otherwise specified. SD, standard deviation; SMA, smooth muscle actin.

## Discussion

Mutations in the *CTNNB1* gene, which encodes the β-catenin protein, is highly prevalent in DF and is considered to contribute to tumour recurrence [16]. Using Sanger sequencing and immunostaining, we detected a positive correlation between *CTNNB1* gene mutation and nuclear β-catenin expression in the DF specimens. It is known that up to 80% of sporadic DF harbour mutations in the *CTNNB1* gene [5]. However, we detected *CTNNB1* mutations in 43 out of 70 patients (61.43%). We assume that the lower positive rate of DF with *CTNNB1* mutation is due to the limitation of biopsied tissues. We included 10 biopsied FFPE tissues in this study, and we cannot exclude the possibility that the biopsied samples might not contain the enough amount of tumor cells for sequencing. We believe that the positive rate of *CTNNB1* mutation could increase if the research was carried out with more biopsied tissues.

We identified a patient harbouring both T41A and S45F mutations in his tumour. The patient was a 48-year-old man with a 4.5-cm sized chest wall mass encasing the rib. Additionally, we detected a T41I mutation in a DF patient, who was a 3-month-old boy with a 5 × 4.8-cm sized scalp mass. A recent study reported that three *CTNNB1* T41I variants were preferentially detected in paediatric DF patients (*n* = 3/22; 13.6%), with only a single instance of DF in an adult (*n* = 1/170; 0.6%) [7].

Among the mutations, the S45F mutation is implicated as a factor that increases the rate of tumour recurrence after excision [2,16–18]. In this study, we reviewed 43 out of 70 DF patients with follow-up data from our institution. However, no significant differences in recurrence were observed between the DF groups with or without *CTNNB1* gene mutations. When we investigated recurrences among DF patients with *CTNNB1* gene mutations, there was no statistical difference between the two groups — DF with T41A or T41I mutation, and DF with S45F or S45P.

We speculated that stabilization and subsequent nuclear localization of β-catenin could be representative of *CTNNB1* mutation status. Indeed, immunostaining results demonstrated that nuclear β-catenin expression was observed more frequently in DF cases with *CTNNB1* mutation than in DF cases without *CTNNB1* mutation. We presented four cases with CTNNB1 mutation which had beta-catenin expression with cytoplasmic/membranous/Golgi staining pattern. The relationship between CTNNB1 gene mutation, β-catenin nuclear localization, and Wnt pathway activation is complex. According to the literatures, nearly half of CTNNB1 mutant cases of endometrial carcinoma had only 5–10% of tumor cells with β-catenin nuclear localization, and the authors suggested that immunohistochemistry could be an initial screen, with CTNNB1 sequencing employed when nuclear localization of β-catenin is absent [9]. Audard et al. report that 37% of tumors with β-catenin mutations they examined were without cytosolic/nuclear staining of β-catenin in hepatocellular carcinoma [19]. Furthermore, *CTNNB1* mutation was observed more frequently in the larger-sized tumours. Therefore, if the excised tumour is small in size, we need to know that β-catenin immunostaining is indicative of the presence of *CTNNB1* mutation, particularly in the case of recurrent tumours. In this study, we divided DF cases into 2 groups according to the specimen types: biopsied tissues and excised tissues. Notably, the correlation between nuclear localization of β-catenin and *CTNNB1* mutation was not identified in biopsied DF tissues. Therefore, we suggest that the *CTNNB1* mutation can be identified in a biopsied specimen even though β-catenin expression is found in the cytoplasm or the membrane and vice versa.

In conclusion, we found that nuclear localization of β-catenin correlated with the *CTNNB1* gene mutation status in DF, and DF demonstrated different clinicopathological features based on the mutations they harbour. We newly identified a patient harbouring both T41A and S45F mutations in his tumour. We demonstrated that *CTNNB1* mutation correlates with nuclear β-catenin expression in larger or excised DF tumours, therefore caution is advised in the interpretation of the immunohistochemical and mutation data when derived from biopsied samples or small tumours.

## Supporting information

S1 Table β-catenin and SMA (smooth muscle actin) expressions in the desmoid-type fibromatosis patients. (DOCX)

S2 Table Clinicopathological findings in desmoid-type fibromatosis patients according to ß-catenin expression and *CTNNB1* mutation status. (DOCX)

S3 Table β-catenin expression and *CTNNB1* mutation in the biopsied and excised tissues of desmoid-type fibromatosis patients. (DOCX)

S1 Dataset (DOCX)

## References

[1] Colombo C, Belfiore A, Paielli N, De Cecco L, Canevari S, Laurini E, et al. beta-Catenin in desmoid-type fibromatosis: deep insights into the role of T41A and S45F mutations on protein structure and gene expression. Mol Oncol. 2017;11(11):1495–507. 10.1002/1878-0261.12101 28627792 PMC5664003

[2] Colombo C, Miceli R, Lazar AJ, Perrone F, Pollock RE, Le Cesne A, et al. CTNNB1 45F mutation is a molecular prognosticator of increased postoperative primary desmoid tumor recurrence: an independent, multicenter validation study. Cancer. 2013;119(20):3696–702. 10.1002/cncr.28271 23913621

[3] Kasper B, Baumgarten C, Garcia J, Bonvalot S, Haas R, Haller F, et al. An update on the management of sporadic desmoid-type fibromatosis: a European Consensus Initiative between Sarcoma PAtients EuroNet (SPAEN) and European Organization for Research and Treatment of Cancer (EORTC)/Soft Tissue and Bone Sarcoma Group (STBSG). Ann Oncol. 2017;28(10):2399–408. 10.1093/annonc/mdx323 28961825 PMC5834048

[4] Mullen JT, DeLaney TF, Rosenberg AE, Le L, Iafrate AJ, Kobayashi W, et al. beta-Catenin mutation status and outcomes in sporadic desmoid tumors. Oncologist. 2013;18(9):1043–9. 10.1634/theoncologist.2012-0449 23960186 PMC3780636

[5] Goldblum JR, Fletcher JA. WHO Classification of Tumours of Soft Tissue and Bone. 4th ed. Bosman FT, Jaffe ES, Lakhani SR, Ohgaki H, editors. Maestro 38330 Saint-Ismier, France: IARC; 2013 2013.

[6] Amary MF, Pauwels P, Meulemans E, Roemen GM, Islam L, Idowu B, et al. Detection of beta-catenin mutations in paraffin-embedded sporadic desmoid-type fibromatosis by mutation-specific restriction enzyme digestion (MSRED): an ancillary diagnostic tool. Am J Surg Pathol. 2007;31(9):1299–309. 10.1097/PAS.0b013e31802f581a 17721184

[7] Trautmann M, Rehkamper J, Gevensleben H, Becker J, Wardelmann E, Hartmann W, et al. Novel pathogenic alterations in pediatric and adult desmoid-type fibromatosis—A systematic analysis of 204 cases. Sci Rep. 2020;10(1):3368. 10.1038/s41598-020-60237-6 32099073 PMC7042250

[8] Bhattacharya B, Dilworth HP, Iacobuzio-Donahue C, Ricci F, Weber K, Furlong MA, et al. Nuclear beta-catenin expression distinguishes deep fibromatosis from other benign and malignant fibroblastic and myofibroblastic lesions. Am J Surg Pathol. 2005;29(5):653–9. 10.1097/01.pas.0000157938.95785.da 15832090

[9] Kim G, Kurnit KC, Djordjevic B, Singh C, Munsell MF, Wang WL, et al. Nuclear beta-catenin localization and mutation of the CTNNB1 gene: a context-dependent association. Mod Pathol. 2018;31(10):1553–9. 10.1038/s41379-018-0080-0 29795437 PMC6168348

[10] Machin P, Catasus L, Pons C, Munoz J, Matias-Guiu X, Prat J. CTNNB1 mutations and beta-catenin expression in endometrial carcinomas. Hum Pathol. 2002;33(2):206–12. 10.1053/hupa.2002.30723 11957146

[11] Tony LN, Allen MG, Todd SB, Maggie CUC, Andy KWC, Dmitry AT, et al. Nuclear beta-catenin in mesenchymal tumors. Mod Pathol. 2005;18(1):68–74. 10.1038/modpathol.3800272 15375433

[12] Gong LH, Liu WF, Ding Y, Geng YH, Sun XQ, Huang XY. Diagnosis and Differential Diagnosis of Desmoplastic Fibroblastoma by Clinical, Radiological, and Histopathological Analyses. Chin Med J (Engl). 2018;131(1):32–6. 10.4103/0366-6999.221274 29271377 PMC5754955

[13] Abraham SC, Reynolds C, Lee JH, Montgomery EA, Baisden BL, Krasinskas AM, et al. Fibromatosis of the breast and mutations involving the APC/beta-catenin pathway. Hum Pathol. 2002;33(1):39–46. 10.1053/hupa.2002.30196 11823972

[14] Liu Q, Fang L, Li B. Desmoid fibromatosis in the foot: A case report and literature review. Medicine (Baltimore). 2018;97(44):e13109. 10.1097/MD.0000000000013109 30383699 PMC6221670

[15] Li Destri G, Ferraro MJ, Calabrini M, Pennisi M, Magro G. Desmoid-type fibromatosis of the mesentery: report of a sporadic case with emphasis on differential diagnostic problems. Case Rep Med. 2014;2014:850180. 10.1155/2014/850180 25349618 PMC4198783

[16] Lazar AJ, Tuvin D, Hajibashi S, Habeeb S, Bolshakov S, Mayordomo-Aranda E, et al. Specific mutations in the beta-catenin gene (CTNNB1) correlate with local recurrence in sporadic desmoid tumors. Am J Pathol. 2008;173(5):1518–27. 10.2353/ajpath.2008.080475 18832571 PMC2570141

[17] Domont J, Salas S, Lacroix L, Brouste V, Saulnier P, Terrier P, et al. High frequency of beta-catenin heterozygous mutations in extra-abdominal fibromatosis: a potential molecular tool for disease management. Br J Cancer. 2010;102(6):1032–6. 10.1038/sj.bjc.6605557 20197769 PMC2844024

[18] van Broekhoven DL, Verhoef C, Grunhagen DJ, van Gorp JM, den Bakker MA, Hinrichs JW, et al. Prognostic value of CTNNB1 gene mutation in primary sporadic aggressive fibromatosis. Ann Surg Oncol. 2015;22(5):1464–70. 10.1245/s10434-014-4156-x 25341748

[19] Madeleine A, Ruediger D, Christian W, Andrea T, Rolf G, Frank G. Correlation between β-catenin mutations and expression of Wnt-signaling target genes in hepatocellular carcinoma. Mol Cancer. 2008;7:21. 10.1186/1476-4598-7-21 18282277 PMC2287186

