# Supplementary material for: Clinicopathological features of 70 desmoid-type fibromatoses confirmed by β-catenin immunohistochemical staining and CTNNB1 mutation analysis
Source: PLoS One. 2021 Apr 29;16(4):e0250619. doi: 10.1371/journal.pone.0250619 (PMC8084228; doi:10.1371/journal.pone.0250619)
Supplement: S1 Table — (DOCX) [file pone.0250619.s001.docx]

**S1 Table. β-catenin and SMA (smooth muscle actin) expressions in the desmoid-type fibromatosis patients**

| Specimen (N = 70) | | SMA Expression | |
| --- | --- | --- | --- |
|  |  | Negative | Positive |
| β-catenin Expression | Negative | 1 | 13 |
|  | Positive | 8 | 48 |
| Fisher's exact test |  | *P* = 0.6755 | |
