# Supplementary material for: Clinicopathological features of 70 desmoid-type fibromatoses confirmed by β-catenin immunohistochemical staining and CTNNB1 mutation analysis
Source: PLoS One. 2021 Apr 29;16(4):e0250619. doi: 10.1371/journal.pone.0250619 (PMC8084228; doi:10.1371/journal.pone.0250619)
Supplement: S2 Table — (DOCX) [file pone.0250619.s002.docx]

**S2 Table. Clinicopathological findings in desmoid-type fibromatosis patients according to ß-catenin expression and *CTNNB1* mutation status**

|  | **IHC- & Mutation-**  **(*n* = 10)** | **IHC+ or Mutation+**  **(*n* = 60)** | ***p-*value** |
| --- | --- | --- | --- |
| **Sex** |  |  | 0.731 |
| **Male** | 5 (50.0) | 24 (40.0) |  |
| **Female** | 5 (50.0) | 36 (60.0) |  |
| **Age at diagnosis (years; mean ± SD)** | 42.2 ± 8.022 | 34.98 ± 2.597 | 0.315 |
| **Site** |  |  | 0.365 |
| **Abdominal wall** | 0 (0.0) | 9 (15.0) |  |
| **Abdominal cavity** | 2 (20.0) | 7 (11.7) |  |
| **Extra-abdominal** | 8 (80.0) | 44 (73.3) |  |
| **Size (cm; mean ± SD)** | 4.2 ± 1.201, n=9 | 7.147 ± 0.5981, n=55 | 0.063 |
| **Specimen** |  |  | >0.999 |
| **Biopsy** | 1 (10.0) | 9 (15.0) |  |
| **Excision** | 9 (90.0) | 51 (85.0) |  |
| **Recurrence (n = 43)** |  |  | 0.618 |
| **Absent** | 3 (75.0) | 21 (53.8) |  |
| **Present** | 1 (25.0) | 18 (16.2) |  |
| **SMA expression** |  |  | >0.999 |
| **Negative** | 1 (10.0) | 8 (13.3) |  |
| **Positive** | 9 (90.0) | 52 (86.7) |  |

Values are expressed as *n* (%) unless otherwise specified. SD, standard deviation; SMA, smooth muscle actin
