# Supplementary material for: Clinicopathological features of 70 desmoid-type fibromatoses confirmed by β-catenin immunohistochemical staining and CTNNB1 mutation analysis
Source: PLoS One. 2021 Apr 29;16(4):e0250619. doi: 10.1371/journal.pone.0250619 (PMC8084228; doi:10.1371/journal.pone.0250619)
Supplement: S3 Table — (DOCX) [file pone.0250619.s003.docx]

**S3 Table. β-catenin expression and *CTNNB1* mutation in the biopsied and excised tissues of desmoid-type fibromatosis patients**

| Specimen (N = 70) | β-catenin Expression (n) | | *CTNNB1* Mutation (n) | |
| --- | --- | --- | --- | --- |
|  | Cytoplasm | Nucleus | No | Yes |
| Biopsy (N = 10) | 1 | 9 | 5 | 5 |
| Excision (N = 60) | 13 | 47 | 22 | 38 |
| Fisher's exact test | *P* = 0.6742 | | *P* = 0.4927 | |
