# Supplementary material for: Clinicopathological features of 70 desmoid-type fibromatoses confirmed by β-catenin immunohistochemical staining and CTNNB1 mutation analysis
Source: PLoS One. 2021 Apr 29;16(4):e0250619. doi: 10.1371/journal.pone.0250619 (PMC8084228; doi:10.1371/journal.pone.0250619)
Supplement: S1 Dataset — (DOCX) [file pone.0250619.s004.docx]

| **No** | **Sex** | **Age** | **nuclear b-catenin** | **Smooth Muscle Actin** | **CTNNB1 Mutation** | **Site** | **Recurrence** | **Procedure** |
| --- | --- | --- | --- | --- | --- | --- | --- | --- |
| 1 | F | 1 | Positive | Positive | T41A | neck | Not evaluated | excision |
| 2 | M | 35 | Positive | Positive | T41A | pleura | No | excision |
| 3 | M | 56 | Positive | Positive | T41A | upper arm | Yes | excision |
| 4 | F | 35 | Positive | Positive | T41A | lower leg | Not evaluated | excision |
| 5 | F | 51 | Positive | Positive | T41A | small bowel | No | excision |
| 6 | F | 14 | Positive | Positive | T41A | thigh | Yes | excision |
| 7 | F | 19 | Positive | Negative |  | lower leg | Not evaluated | excision |
| 8 | M | 11 | Positive | Positive | T41A | inguinal area | No | excision |
| 9 | M | 58 | Positive | Positive |  | inguinal area | No | biopsy |
| 10 | F | 55 | Positive | Negative |  | chest wall | No | biopsy |
| 11 | F | 10 | Positive | Positive | T41A | wrist | Yes | excision |
| 12 | F | 33 | Positive | Positive | S45P | abdominal wall | Not evaluated | excision |
| 13 | M | 42 | Positive | Positive |  | abdominal wall | No | biopsy |
| 14 | F | 6 | Positive | Positive | T41A | knee | Not evaluated | excision |
| 15 | F | 38 | Positive | Positive | T41A | retroperitoneum | No | excision |
| 16 | F | 45 | Positive | Positive |  | peritibial | Yes | excision |
| 17 | M | 13 | Positive | Positive |  | thigh | Not evaluated | excision |
| 18 | M | 14 | Positive | Positive | T41A | popliteal area | No | excision |
| 19 | M | 11 | Positive | Positive | T41A | thigh | Yes | excision |
| 20 | F | 38 | Negative | Negative |  | retroperitoneum | Not evaluated | excision |
| 21 | F | 77 | Negative | Positive | S45F | chest wall | No | excision |
| 22 | F | 19 | Positive | Positive | S45F | neck | Yes | biopsy |
| 23 | F | 30 | Positive | Positive |  | abdominal wall | No | excision |
| 24 | M | 48 | Positive | Positive | T41A | chest wall | Not evaluated | excision |
| 25 | M | 68 | Positive | Positive | T41A | mesentery | No | excision |
| 26 | M | 47 | Negative | Positive |  | eyelid | Not evaluated | excision |
| 27 | M | 62 | Positive | Positive | T41A | mesentery | No | biopsy |
| 28 | M | 59 | Positive | Positive | T41A | mesentery | No | excision |
| 29 | M | 17 | Positive | Positive | T41A | forearm | Yes | excision |
| 30 | F | 39 | Positive | Positive |  | back/inguinal | Yes | excision |
| 31 | F | 36 | Positive | Positive |  | abdominal wall | No | biopsy |
| 32 | F | 36 | Positive | Positive | S45P | abdominal wall | No | excision |
| 33 | F | 23 | Positive | Positive | S45F | thigh | Yes | excision |
| 34 | M | 69 | Positive | Negative | T41A | mesentery | Not evaluated | excision |
| 35 | M | 33 | Negative | Positive | T41A | upper arm | Not evaluated | excision |
| 36 | F | 78 | Positive | Negative |  | nasal cavity | Not evaluated | excision |
| 37 | M | 18 | Positive | Positive | S45F | abdominal wall | Yes | excision |
| 38 | F | 24 | Positive | Positive | S45F | mediastinum | Yes | biopsy |
| 39 | F | 19 | Positive | Positive | T41A | thigh | Yes | excision |
| 40 | M | 3 | Positive | Negative |  | wrist | Not evaluated | excision |
| 41 | M | 65 | Positive | Positive |  | hand | Not evaluated | excision |
| 42 | F | 32 | Positive | Positive | T41A | chest wall | Not evaluated | biopsy |
| 43 | F | 59 | Negative | Positive |  | Breast | No | biopsy |
| 44 | F | 36 | Positive | Positive | S45F | abdominal wall | Not evaluated | excision |
| 45 | M | 48 | Positive | Negative | T41A,S45F | rib | Not evaluated | excision |
| 46 | F | 32 | Negative | Positive | S45F | popliteal area | Yes | excision |
| 47 | F | 51 | Positive | Negative |  | muscle | Not evaluated | excision |
| 48 | F | 14 | Positive | Positive | S45F | abdominal wall | Yes | excision |
| 49 | F | 38 | Positive | Positive | S45F | neck | Yes | excision |
| 50 | F | 26 | Positive | Negative | T41A | shoulder | No | excision |
| 51 | M | 30 | Positive | Positive | T41A | upper arm | No | excision |
| 52 | M | 39 | Positive | Positive | T41A | back | Not evaluated | excision |
| 53 | F | 74 | Positive | Positive | S45F | neck | Not evaluated | biopsy |
| 54 | M | 49 | Negative | Positive |  | hand | Not evaluated | excision |
| 55 | F | 12 | Positive | Positive | T41A | thigh | Yes | excision |
| 56 | M | 0 | Positive | Positive | S45F | scalp | No | excision |
| 57 | M | 0 | Negative | Positive |  | preauricular area | Not evaluated | excision |
| 58 | F | 52 | Negative | Positive |  | small bowel | No | excision |
| 59 | F | 84 | Negative | Positive |  | peritoneum | Not evaluated | excision |
| 60 | F | 31 | Positive | Positive | T41A | foot | Not evaluated | excision |
| 61 | M | 1 | Negative | Positive |  | perinasal area | Not evaluated | excision |
| 62 | M | 49 | Positive | Positive |  | mesentery | No | excision |
| 63 | M | 12 | Positive | Positive |  | thigh | No | excision |
| 64 | F | 31 | Positive | Positive |  | inguinal area | Yes | excision |
| 65 | F | 42 | Negative | Positive | S45F | chest wall | Not evaluated | excision |
| 66 | F | 35 | Positive | Positive | T41A | abdominal wall | No | excision |
| 67 | M | 52 | Negative | Positive |  | hand | No | excision |
| 68 | F | 40 | Negative | Positive |  | vulva | Yes | excision |
| 69 | F | 65 | Positive | Positive |  | sole | Yes | excision |
| 70 | F | 32 | Positive | Positive | T41A | abdominal wall | Not evaluated | excision |
